# Comparative outcome assessment of epidermal growth factor receptor tyrosine kinase inhibitors for the treatment of advanced non-small-cell lung cancer: a network meta-analysis

**DOI:** 10.18632/oncotarget.23668

**Published:** 2017-12-23

**Authors:** Ramon Andrade De Mello, Carles Escriu, Pedro Castelo-Branco, Paloma Lucena Cabral, Giannis Mountzios, Gilberto de Lima Lopes, Pedro Madureira

**Affiliations:** ^1^ Division of Oncology, School of Medicine, Department of Biomedical Sciences and Medicine, University of Algarve, Faro, Portugal; ^2^ Algarve Biomedical Center, Campus Gambelas, Faro, Portugal; ^3^ Research Centre/Department of Medical Oncology, Haroldo Juaçaba Hospital, Ceará Cancer Institute, Fortaleza, CE, Brazil; ^4^ Department of Medical Oncology, The Clatterbridge Cancer Centre NHS Foundation Trust, Warrington, Wirral and Liverpool, Merseyside, United Kingdom; ^5^ Cancer Research Centre, Department of Molecular and Clinical Cancer Medicine, The University of Liverpool, Liverpool, United Kingdom; ^6^ Centre for Biomedical Research, University of Algarve, Faro, Portugal; ^7^ Special Training Program (PET), Faculty of Medicine, Federal University of Ceará, Fortaleza, CE, Brazil; ^8^ Department of Medical Oncology, University of Athens, Athens, Greece; ^9^ Sylvester Comprehensive Cancer Centre at the University of Miami, Miami, FL, USA; ^10^ Institute for Molecular and Cell Biology (IBMC) and Institute for Investigation and Innovation in Health (i3S), University of Porto, Porto, Portugal

**Keywords:** non-small cell lung cancer, epidermal growth factor receptor, tyrosine kinase inhibitors

## Abstract

**Introduction:**

Tyrosine kinase inhibition of the epidermal growth factor receptor (EGFR) is the standard in the first line treatment of patients with advanced non-small–cell lung cancer (NSCLC) harbouring EGFR activating mutations. Here we aim to discern efficacy and toxicity measures through a meta-analysis of published studies that could aid treatment selection.

**Materials And Methods:**

We performed a meta-analysis of the main randomized clinical trials evaluating the currently approved EGFR-TKIs in first-line of treatment of EGFR-positive advanced NSCLC. Cochrane guidelines were used for statistical analysis.

**Results:**

3,179 patients were included. All EGFR TKIs showed improved outcomes with respect to ORR and PFS when compared to standard platinum-doublet chemotherapy. Comparative ORR for gefitinib, erlotinib and afatinib were 52.1%, 67.3% and 61.6% respectively. HRs for PFS were 0.62 (95% CI, 0.38–1.00) for gefitinib, 0.28 (95% CI, 0.17–0.45) for erlotinib and 0.40 (95% CI, 0.20–0.83) for afatinib. HRs for OS were not statistically significant for any agent.

**Conclusions:**

Our results suggest similar clinical efficacy and higher toxicity of Afatinib treatment. As this still remains the agent with best CSF penetration, we suggest its use is limited to patients presenting with brain metastasis. We suggest the use of Gefitinib in patients without CNS involvement. Faced with the impossibility to dose-reduce Gefitinib, Erlotinib represents a tolerable and effective alternative to Afatinib and Gefitinib if response to EGFR inhibition is considered still effective.

## INTRODUCTION

Non-small-cell lung cancer (NSCLC) is the major cause of cancer-related death worldwide [1]. The Epidermal Growth Factor Receptor (EGFR), a transmembrane glycoprotein, is mutated in approximately 10–15% of European patients, more frequently in women, adenocarcinoma type and never-smokers [2]. When the *EGFR* gene is mutated, (most commonly with exon 19 deletions or exon 21 L858R point mutation), constitutive receptor activation influences the cell cycle, the apoptotic pathway and the production of inflammatory agents [3]. This understanding of EGFR signalling led to the development of specific tyrosine-kinase inhibitors (TKIs) [4], which reached three generations: gefitinib and erlotinib (first); afatinib, dacomitinib, and neratinib (second); rociletinib, HM61713, osimertinib and others (third). The last generation overcomes the threonine-to-methionine substitution (T790M) in exon 20 of the EGFR gene, responsible for 50% of resistance mechanisms to first line anti-EGFR therapy with first and second generation agents [5]. Only gefitinib, erlotinib, and afatinib are approved by Food and Drug Administration (FDA) thus far for the first line setting [6–8].

In patients whose tumours harbours an activating *EGFR* mutation, EGFR TKIs should be used as first-line therapy [6–9], whereas for the rest of NSCLC cases, standard treatment currently consists of platinum-based doublet chemotherapy. Gefitinib, erlotinib and afatinib show higher response rates and longer progression free survival than chemotherapy in those patients, as tested in several clinical trials exhibiting consistent results [10–20], all of them favouring the target therapy.

Since there are several similar drugs targeting the *EGFR* mutation in NSCLC first line setting, the critical question emerging is which one should be best for this setting. Our analysis presents the findings of a network meta-analysis, attempting to access the main outcomes among EGFR TKIs in NSCLC, exploiting the data of clinical trials with gefitinib, erlotinib and afatinib. Recently, the Lux-Lung 7 study reported longer PFS and similar OS when comparing Afatinib with Gefitinib, but a triple arm comparison of all these agents is unlikely to occur. Here we aimed to provide an indirect comparison among these drugs which may contribute to guide the drug choice for physicians.

## MATERIALS AND METHODS

For this comparative meta-analysis, we performed computerized searches of the Medline. Embase, Scopus and Information Sciences Institute (ISI) databases up to August 14, 2016, using the following terms: “gefitinib” OR “afatinib” OR “erlotinib” AND “NSCLC” OR “lung cancer” OR “epidermal growth factor”. These searches were complemented by examining review articles. Only articles published in English, available in full text and reporting results of randomized, double-arm, phase III clinical trials comparing EGFR-TKIs with chemotherapy regimens were included. The most recent –updated- data of the studies were used for the meta-analysis. For gefitinib, erlotinib and afatinib, only first line treatments were considered due to the paucity of trials comparing these agents to chemotherapy in second line. There were no time restrictions in the search. Exclusion criteria were: trials with patients presenting Eastern Cooperative Oncology Group (ECOG) performance status > 2 and those including EGFR TKI plus chemotherapy versus chemotherapy (Effectiveness of EGFR-TKIs may be obscured in this setting). Case reports or patient series, which report few patients, were excluded. All abstracts were screened twice and unrelated studies were excluded.

For included trials, we extracted data on: title, first author, year of publication, study design (inclusion and exclusion criteria), patient’s characteristics (median patient age, stage of disease, performance status, gender, smoking status, histology, tissue-assessed EGFR mutation), treatment schedules and line of treatment, outcomes from the trial, incidence of adverse events, demographic data. If the study was updated, main outcomes were extracted from the last published article. Data extraction was done independently by two of the authors and divergences were resolved by consensus with a third author.

The primary outcome of this meta-analysis was objective response rate (ORR). Second outcomes were progression free survival (PFS), overall survival (OS) and incidence of adverse events (AE). Summary measures were risk ratio (95% confidence interval [CI]; 95% PI) for ORR and AE and hazard ratio for OS and PFS.

ORR was defined as the proportion of patients who presented complete or partial response, assessed by Response Evaluation Criteria in Solid Tumours (RECIST) [21] in most of the studies. The time of assessment varied for each trial. PFS was the time, in months, from the randomization until disease progression, or death. OS was the time, in months, from the randomization to death. AE could be any unfavourable and unintentional sign, symptom, or disease temporarily associated with the use of the drugs, without any judgment about causality or relationship to them. Relevant adverse events of all grades related by two or more studies were condensed by each EGFR TKI arm and compared as meta-estimation with another EGFR TKI.

Statistical analysis was directed by Cochrane Guidelines [22]. We combined the risk ratios from each study using the random-effects model (Mantel-Haenzsel) [23]. For the hazard ratios, the Inverse Variance method was used. The heterogeneity between trials was estimated by the *I^2^* statistic. We used the Review Manager version 5.3.5.

## RESULTS

**Figure 1:**
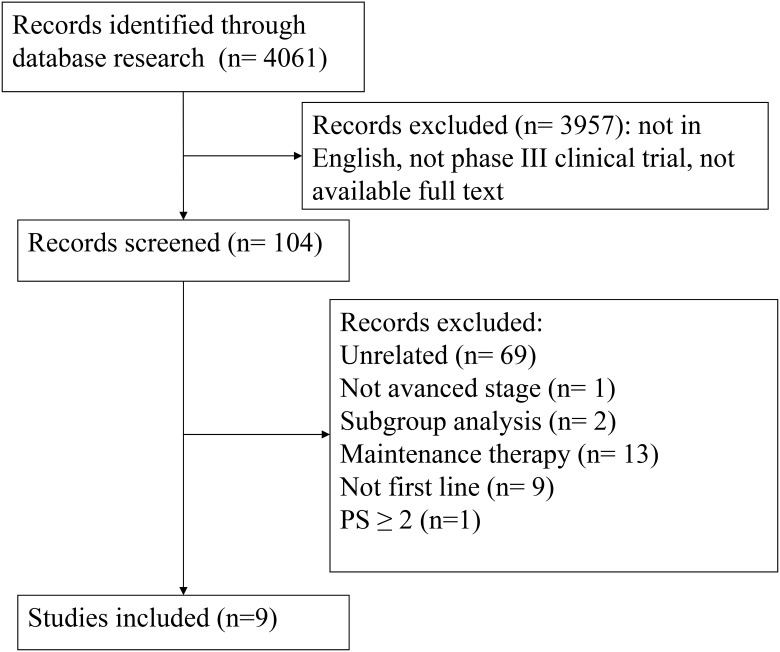
Study selection

As shown in the flow chart of the meta-analysis (Figure 1), 09 eligible studies were identified. All of them were included in the current analysis (Table 1), totalizing 3,179 patients. NEJ002 [15]; WJTOG3405 [16], First-SIGNAL [17], and IPASS [14] evaluated gefitinib as first-line treatment to, respectively, carboplatin plus paclitaxel, cisplatin plus docetaxel, cisplatin plus gemcitabine, and carboplatin plus paclitaxel; LUX-Lung 3 [24] and LUX-Lung 6 [19] compared afatinib as first-line treatment with cisplatin plus pemetrexed and gemcitabine, respectively. EURTAC [20], OPTIMAL [12] and ENSURE [25] compared first-line erlotinib with cisplatin plus docetaxel, gemcitabine plus carboplatin, and cisplatin plus gemcitabine, respectively. NEJ002, IPASS, and OPTIMAL published updated outcomes, so 12 reports were used in total for this meta-analysis.

**Table 1:** Patient demographics and disease characteristics of included studies

Study	First author	Population	Line	Treatment arms	Response criteria
ENSURE (2015)	Wu	Chemotherapy-naïve patients from China, Malaysia, and the Philippines with stage IIIB/IV EGFR mutation-positive NSCLC	First	Erlotinib 150 mg/day (*n =* 110) Gemcitabine 1000 mg/m^2^plus cisplatin 75 mg/m^2^ every 3 weeks (*n =* 107)	RECIST
LUX-Lung 6 (2014)	Wu	Patients with previously untreated stage IIIB or IV lung adenocarcinoma and EGFR mutation-positives	First	Afatinib 40 mg/day (*n =* 242) Gemcitabine 1000 mg/m^2^plus cisplatin 75 mg/m^2^ every 3 weeks (*n =* 122)	RECIST
LUX-Lung 3 (2013)	Sequist	Treatment-naïve patients with advanced lung adenocarcinoma and EGFR mutation-positives	First	Afatinib 40 mg/day (*n =* 230) Cisplatin 75 mg/m^2^ and pemetrexed 500 mg/m^2^ (*n =* 115)	RECIST
EURTAC (2012)	Rosell	European patients with stage IIIB or IV NSCLC and EGFR mutations who had no history of chemotherapy for metastatic disease	First	Erlotinib 150 mg/day (*n =* 86) Cisplatin 75 mg/m^2^ plus docetaxel 75 mg/m^2^ or cisplatin 75 mg/m^2^ plus gemcitabine 1250 mg/m^2^ (*n=* 87)	RECIST
First-SIGNAL (2012)	Han	Chemotherapy-naïve and never-smokers patients with stage IIIB or IV adenocarcinoma of the lung	First	Gefitinib 250 mg/day (*n =* 159) Gemcitabine 1,250 mg/m^2^ plus cisplatin 75 mg/m^2^ every 3 weeks (*n =* 150)	WHO
OPTIMAL (2011)	Zhou and Wu	Chinese patients with stage IIIB or IV NSCLC and a confirmed activating mutation of EGFR, without receiving therapy for metastatic disease	First	Erlotinib 150 mg/day (*n =* 82) Gemcitabine 1000 mg/m^2^plus carboplatin AUC = 5 every 3 weeks (*n =* 72)	RECIST
NEJ002 (2010)	Maemondo	Japanese patients with metastatic NSCLC and EGFR mutations who had not previously received chemotherapy	First	Gefitinib 250 mg/day (*n =* 114) Carboplatin AUC = 6 plus paclitaxel 200 mg/m^2^ every 3 weeks (*n =* 114)	RECIST
WJTOG3405 (2009)	Mitsudomi	Patients with advanced or recurrent NSCLC harbouring an activating mutation of the EGFR	First	Gefitinib 250 mg/day (*n =* 88) Cisplatin 80 mg/m^2^ plus docetaxel 60 mg/m^2^ (*n =* 89)	RECIST
IPASS (2009)	Mok	Asian, nonsmokers or light smokers patients with stage IIIB or IV adenocarcinoma of the lung who had no previous chemotherapy	First	Gefitinib 250 mg/day (*n =* 609) Carboplatin AUC = 5 or 6 plus paclitaxel 200 mg/m^2^ every 3 weeks (*n =* 608)	RECIST

Abbreviations: NSCLC, non-small-cell lung cancer; EGFR, epidermal growth factor receptor; AUC, area under the curve; RECIST, response evaluation criteria in Solid tumors; WHO, world health organization.

Patients’ characteristics are summarized in Table 2. More patients were female (2,315 of 3,179 [72.8%]), never smokers (2,606 of 3,179 [81.9%]), with performance status from 0 to 1 (2,974 of 3,179 [93.5%]) and had tumours of adenocarcinoma histology (3,068 of 3,179 [96.5%]). Disease stage was not summarized because of differences in evaluation among studies.

**Table 2:** Patient demographics and disease characteristics of included studies

Characteristic		Gefitinib (*n* = 968)	Control (*n* = 958)	Erlotinib (*n* = 278)	Control (*n* = 266)	Afatinib (*n* = 472)	Control (*n* = 237)
Sex	Male	213 (22%)	210 (21.9%)	104 (37.4%)	90 (33.8%)	170 (36%)	77 (32.5%)
	Female	755 (78%)	748 (78.1%)	174 (62.6%)	176 (66.2%)	302 (64%)	160 (67.5%)
Age (median)†		60.5	60	59.8	60	59.7	59.5
Smoking status	Never smoker	866 (89.5%)	842 (87.9%)	195 (70.1%)	187 (70.3%)	336 (71.2%)	180 (75.9%)
	Previous or current smoker	102 (10.5%)	116 (12.1%)	140 (50.4%)	79 (29.7%)	136 (28.8%)	57 (24.1%)
ECOG	0–1	892 (92.1%)	877 (91.5%)	252 (90.6%)	245 (92.1%)	472 (100%)	236 (99.6%)
	2	76 (7.9%)	81 (8.5%)	26 (9.4%)	21 (7.9%)	0 (0%)	1 (0.4%)
Histologic diagnosis	Adenocarcinoma	926 (95.7%)	934 (97.5%)	258 (92.8%)	241 (90.6%)	472 (100%)	237 (100%)
	Other	39 (4%)	20 (2.1%)	20 (7.2%)	25 (9.4%)	0 (0%)	0 (0%)
	Unknown	3 (0.3%)	3 (0.3%)	0 (0%)	0 (0%)	0 (0%)	0 (0%)
EGFR mutation	Positive	358 (37%)	345 (36%)	278 (100%)	266 (100%)	472 (100%)	237 (100%)
	Negative	118 (12.2%)	112 (11.7%)	0 (0%)	0 (0%)	0 (0%)	0 (0%)
	Unknown	492 (50.8%)	501 (52.3%)	0 (0%)	0 (0%)	0 (0%)	0 (0%)

Abbreviations: ECOG, Eastern Collaborative Oncology Group; EGFR, epidermal growth factor receptor.

**Figure 2:**
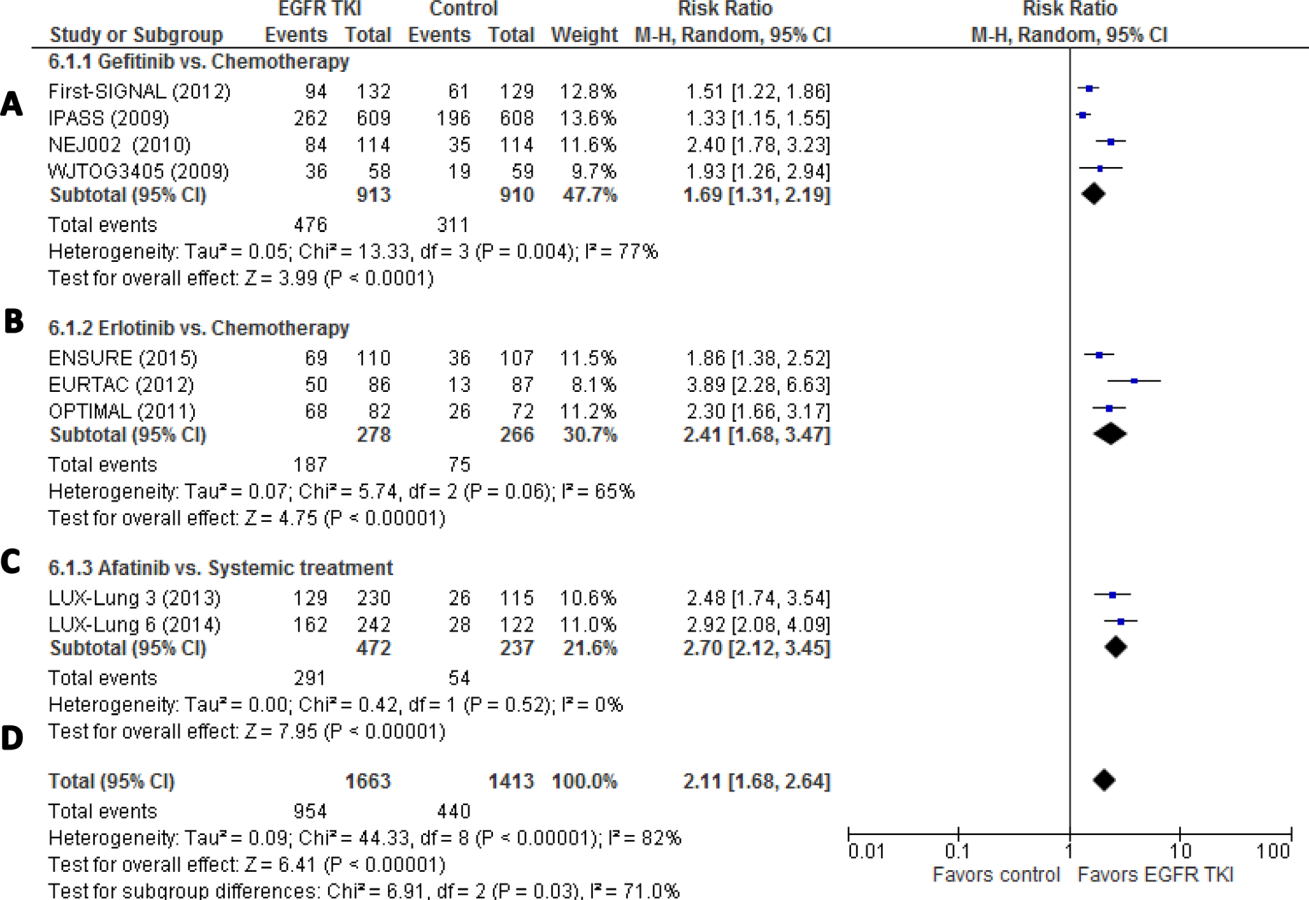
(**A–D**) Individual study and meta-estimate risk ratio of objective response ratio for gefitinib, afatinib, and erlotinib. ORR, overall response rate; PFS, progression-free-survival; OS, overall survival.

The risk ratio of objective response rate (ORR) is shown in Figure 2. For gefitinib versus chemotherapy as first-line treatment, 52.1% (476 out of 913) of patients treated with gefitinib showed complete or partial response against 34.2% (311 out of 910) of patients treated with chemotherapy, and the pooled risk ratio (RR) was 1.69 (95% CI, 1.31–2.19; *p* < 0.0001). For afatinib versus chemotherapy, RR was 2.70 (95% CI, 2.12–3.45; *p* < 0.0001); 61.6% (291 of 472) of patients in the afatinib arm had response, compared to 22.8% (54 out of 237) in the chemotherapy arm. For erlotinib versus chemotherapy, RR was 2.41 (95% CI, 1.68–3.47; *p* < 0.0001). ORR was 67.3% (187 patients of 278 for the erlotinib arm and 28.2% (75 out of 266) for the chemotherapy arm. Heterogeneity was high between studies (*I*^2^ = 78%).

**Figure 3:**
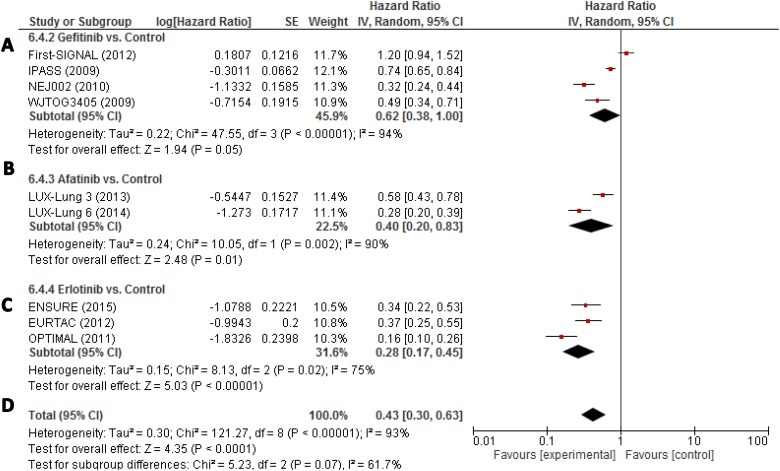
(**A–D**) Individual study hazard ratios with pooled estimation for progression-free survival for gefitinib, erlotinib, and afatinib. ORR, overall response rate; PFS, progression-free-survival; OS, overall survival.

In terms of progression free survival (PFS), the pooled hazard ratio (HR) for gefitinib as first-line HR was 0.62 (95% CI, 0.38–1.00 (Figure 3). In the afatinib analysis, HR was 0.40 (95% CI, 0.20–0.83). In the erlotinib one, HR was 0.28 (95% CI, 0.17–0.45). Heterogeneity was high (*I*^2^ = 93%).

**Figure 4:**
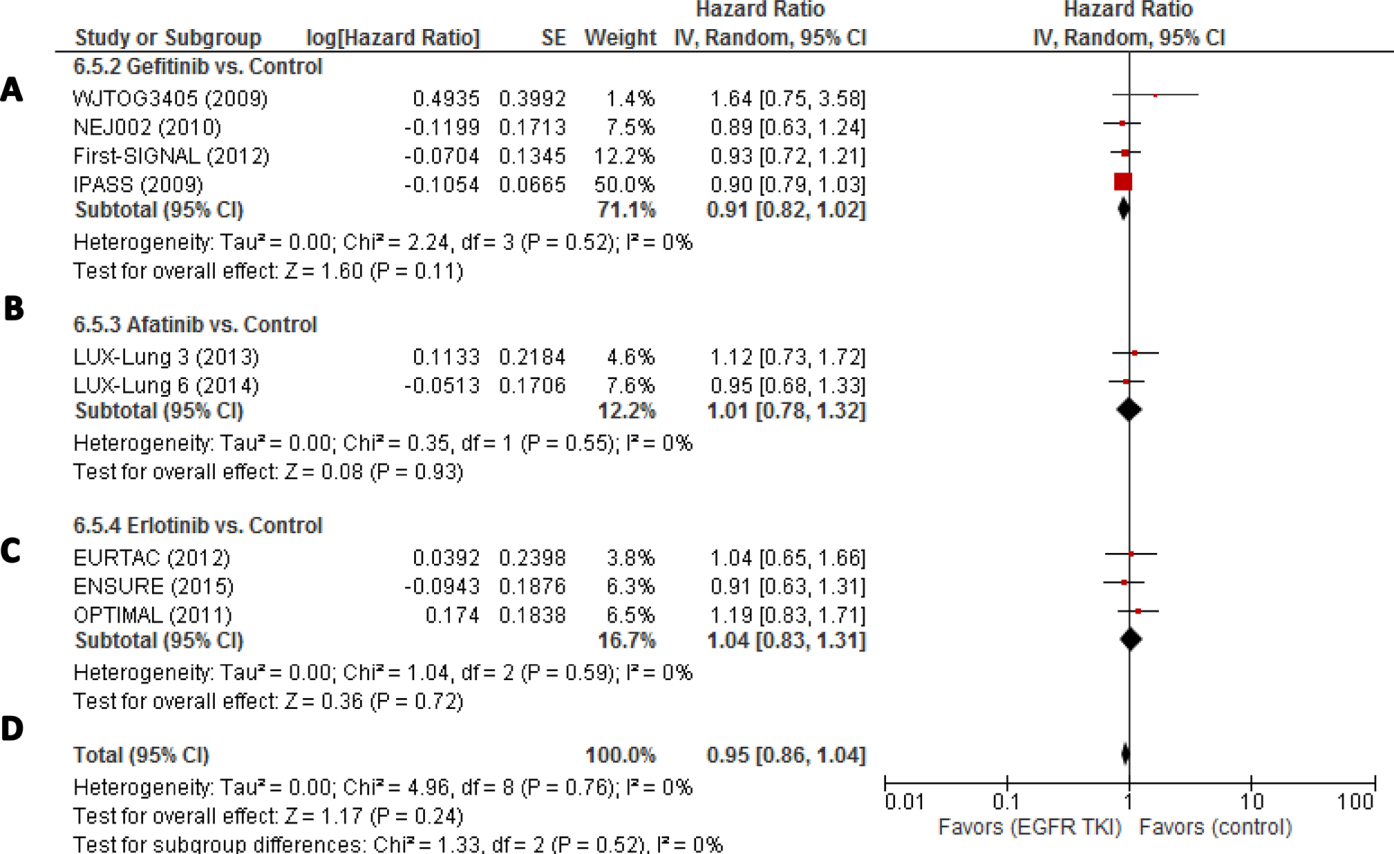
(**A–D**) Individual study hazard ratios with pooled estimation for overall survival for gefitinib, erlotinib, and afatinib. ORR, overall response rate; PFS, progression-free-survival; OS, overall survival.

Accessing overall survival (OS), heterogeneity was very low (*I*^2^ = 0%). For Gefitinib HR was 0.91 (95% CI, 0.82–1.02; *p* = 0.11) (Figure 4). For afatinib, HR was 1.01 (95% CI, 0.78–1.32; *p* = 0.93) and 1.04 (95% CI, 0.83–1.31; *p* = 0.72) for erlotinib.

**Figure 5:**
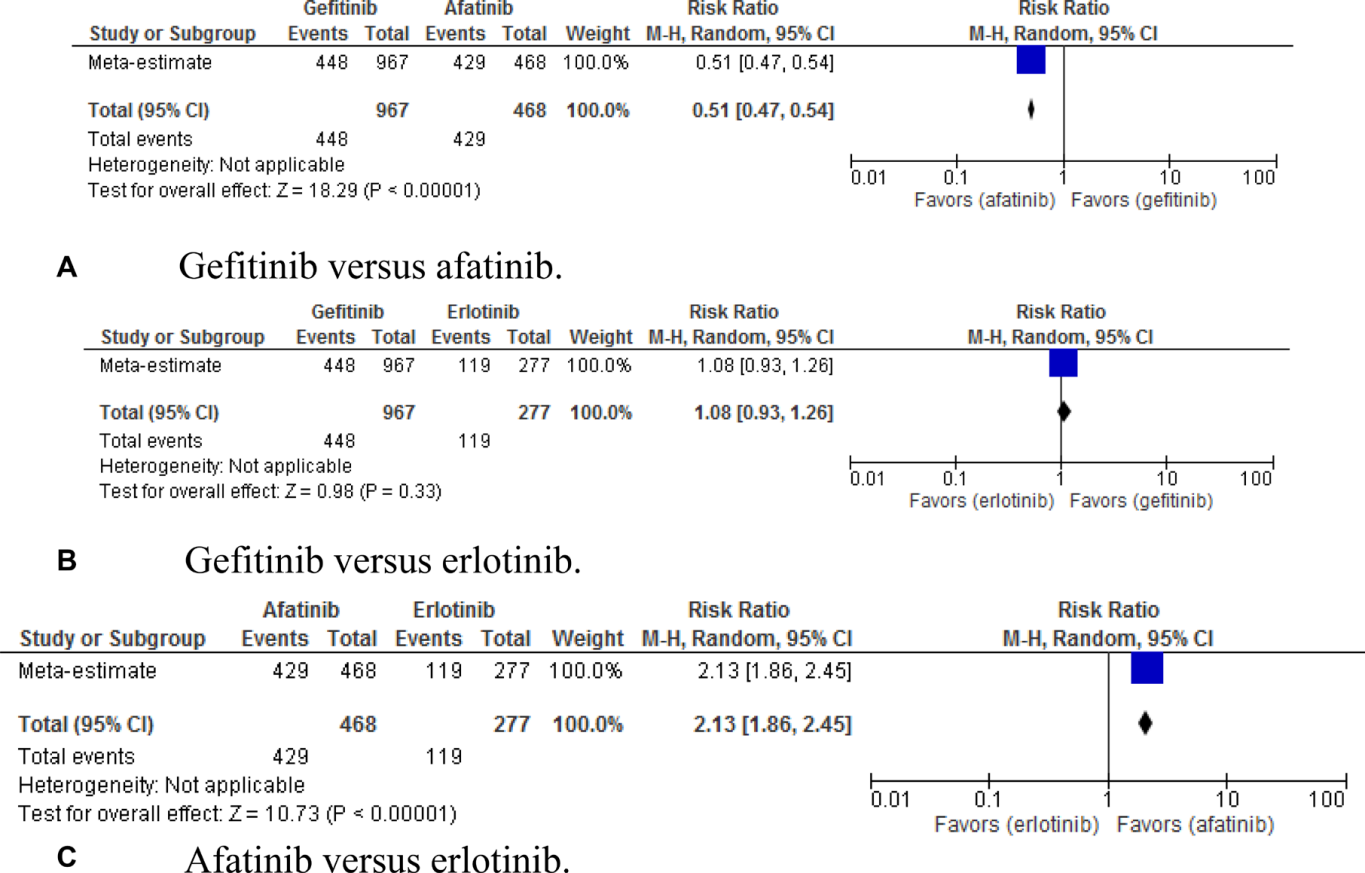
(**A–C**) Pooled risk ratio of gefitinib, erlotinib, and afatinib indirectly compared for the ocurrence of diahrrea. EGFR, epidermal growth factor receptor; ORR, overall response rate; PFS, progression-free-survival; OS, overall survival.

**Figure 6:**
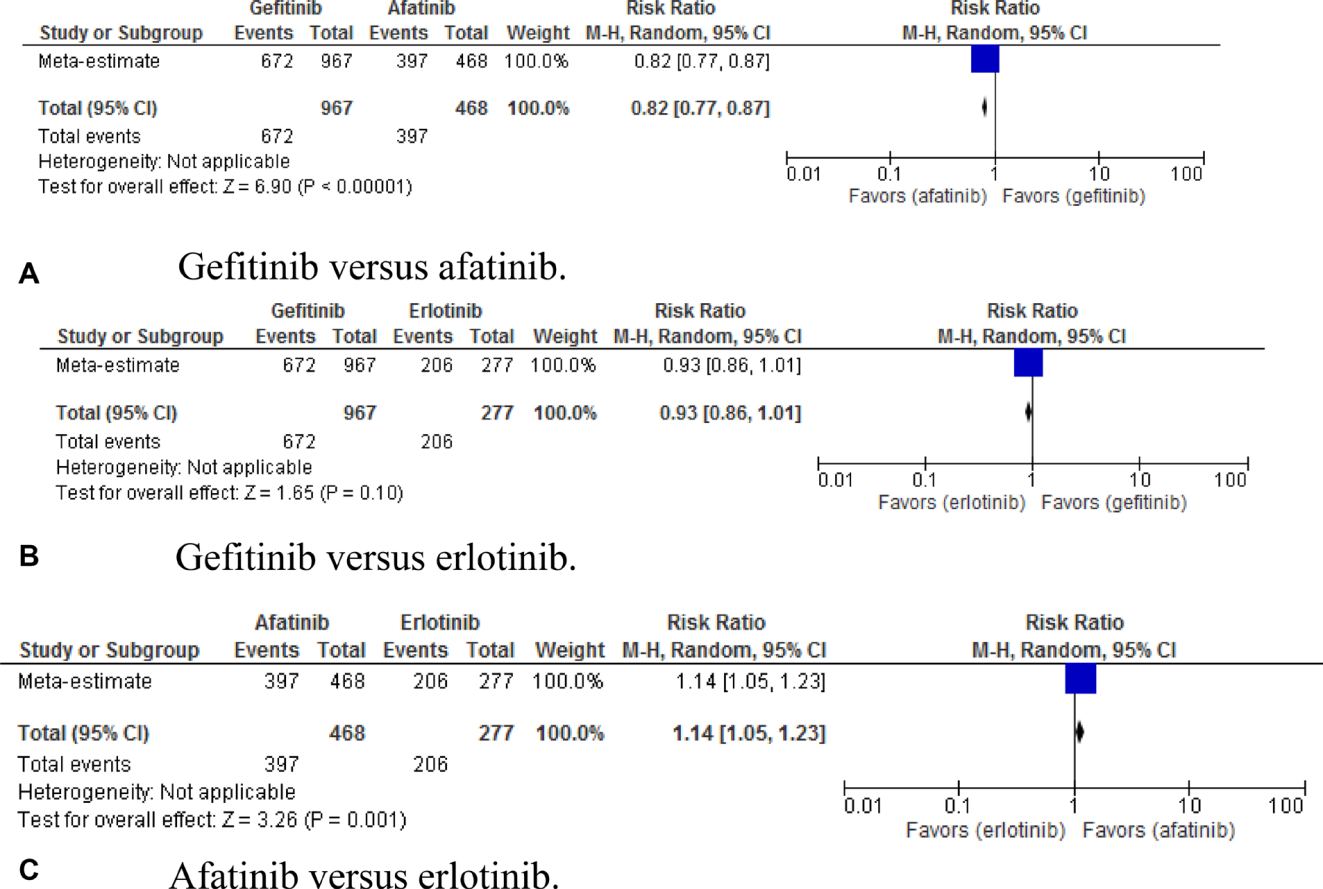
(**A–C**) Pooled risk ratio of gefitinib, erlotinib, and afatinib indirectly compared for the ocurrence of skin rash.

**Figure 7:**
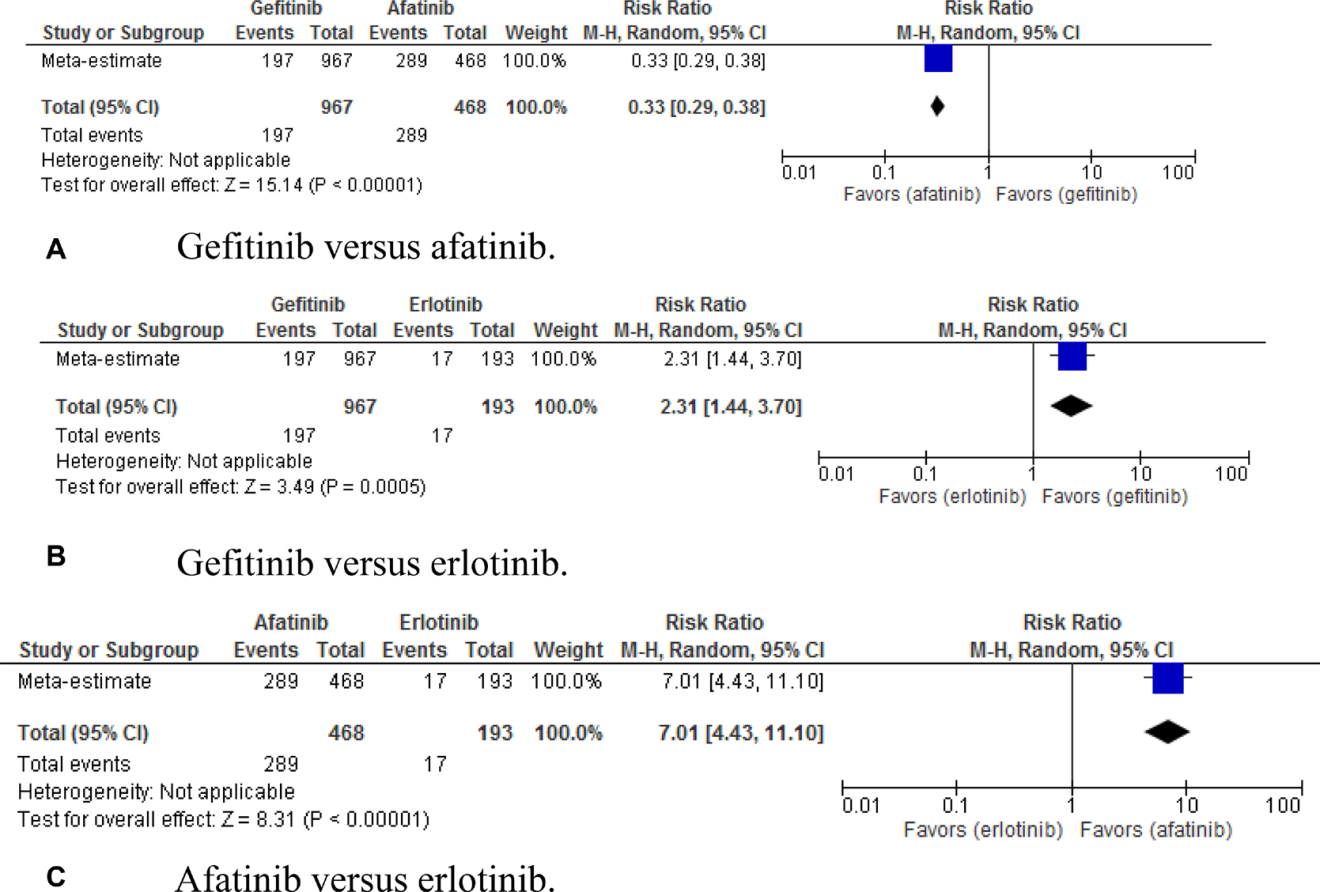
(**A–C**) Pooled risk ratio of gefitinib, erlotinib, and afatinib indirectly compared for the ocurrence of stomatitis.

**Figure 8:**
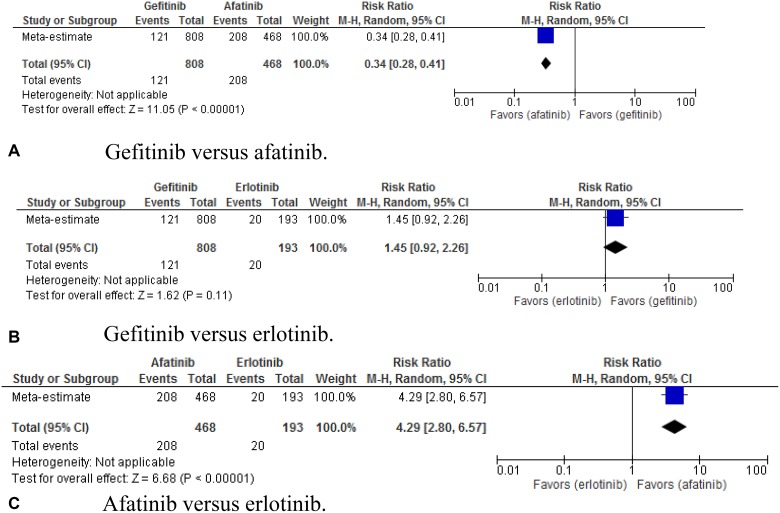
(**A–C**) Pooled risk ratio of gefitinib, erlotinib, and afatinib indirectly compared for the ocurrence of paronychia.

Most common adverse events of EGFR TKIs [26] were analysed (Figures 5–8). Diarrhoea of any grade was a common side effect for these patients. Comparing gefitinib with afatinib, RR was 0.51 (95% CI, 0.47–0.54; *p* < 0.00001); gefitinib with erlotinib, RR was 1.00 (95% CI, 0.93–1.26; *p* = 0.03); and afatinib with erlotinib, RR was 2.13 (95% CI, 1.86–2.45; *p* < 0.00001).

The incidence of skin rash was also observed. In the indirect comparison, gefitinib versus afatinib showed RR of 0.82 (95% CI, 0.77–0.87; *p* < 0.00001). For gefitinib versus erlotinib, RR was 0.93 (95% CI, 0.86–1.01; *p* = 0.10). For afatinib versus erlotinib, RR was 1.14 (95% CI, 1.05 – 1.23; *p* = 0.001) (Figure 6).

For the occurrence of stomatitis (Figure 7), the pooled RR for gefitinib versus afatinib was 0.33 (95% CI, 0.29–0.38; *p* < 0.00001); gefitinib versus erlotinib, 2.31 (95% CI, 1.44–3.70; *p* = 0.00015); afatinib versus erlotinib, 7.01 (95% CI, 4.43–11.10; *p* < 0.00001).

Paronychia was also accessed (Figure 8). The indirect comparison showed RR of 0.34 (95% CI, 0.28–0.41; *p* < 0.00001) for gefitinib versus afatinib, 1.45 (95% CI, 0.92–2.26; *p* = 0.11) for gefitinib versus erlotinib, and 4.29 (95% CI, 2.80–6.57; *p* < 0.00001) for afatinib versus erlotinib.

## DISCUSSION

Currently, the landscape of NSCLC treatment is changing. Most recently, the use of EGFR TKI agents for patients harbouring activating mutations of *EGFR* (exons 18–21) is the standard of care. Several drugs have been approved in this setting, including gefitinib, erlotinib and recently afatinib. In this meta-analysis, gefitinib, erlotinib, and afatinib were superior in terms of objective response rate and progression free survival than platinum-based chemotherapy, but, as expected due to the cross-over effect, there was no statistically significant differences in terms of OS for either of the three drugs. Overall, gefitinib had the most consistent efficacy profile from a statistical point of view, and erlotinib had the best efficacy profile in terms of comparative improvement of PFS.

Our results challenge the recently reported results of LUX-Lung 7 [27], a phase 2b trial comparing afatinib with gefitinib as first-line treatment in patients harboring *EGFR* mutations, that showed improvement in PFS and ORR with afatinib over gefitinib. Nevertheless, previous meta-analysis [28–30] evaluating first-line therapies of EGFR TKIs in EGFR mutation positive patients had not confirmed the results of this study. Although LUX-LUNG 7 is the only prospective, randomized clinical trial, it also harboured several drawbacks, including the small number of events, the lack of statistical power and the three co-primary endpoints. Our meta-analysis, on the other hand, is a retrospective collective analysis of data, but includes a large number of patients and possesses robust statistical power.

Afatinib was more likely to be related to adverse events, as expected because of its irreversible binding to ATP site of EGFR, HER2 and HER4, in contrast to the reversible nature of binding of gefitinib and erlotinib. [31–32]. Differences between gefitinib and erlotinib were not statistically significant, except for paronychia, which was more frequent with erlotinib.

Limitations of our study include its retrospective nature and the indirect comparison between gefitinib, erlotinib and afatinib, since there is a paucity of head-to-head clinical trials, with the exception of LUX-LUNG 7; the high heterogeneity obtained during the data analysis; and the relative paucity of studies evaluating afatinib. Strengths of our study included the large number of patients, the robust statistical design and the broader range of therapies included, as we present data on the three approved first-line drugs.

Future studies are warranted to associate each type of EGFR-activating mutation to the efficacy of a specific treatment and to compare new drugs, as osimertinib, with first and second generation TKIs.

In conclusion, gefitinib, erlotinib, and afatinib are effective in the treatment of NSCLC in terms of progression free survival and objective response rate. Gefitinib had the most consistent efficacy profile from a statistical point of view, and erlotinib had the best efficacy profile in terms of comparative improvement of PFS. As Afatinib still remains the agent with best CSF penetration, we suggest its use is limited to patients presenting with brain metastasis. We suggest the use of Gefitinib in patients without CNS involvement. Faced with the impossibility to dose-reduce Gefitinib, Erlotinib represents a tolerable and effective alternative to Afatinib and Gefitinib if response to EGFR inhibition is considered still effective.
